# Comparison of sedentary time, number of steps and sit-to-stand-transitions of desk-based workers in different office environments including working from home: analysis of quantitative accelerometer data from the cross-sectional part of the SITFLEX Study

**DOI:** 10.5271/sjweh.4228

**Published:** 2025-07-01

**Authors:** Martha Sauter, Eva Backé, Carina Pfab, Michaela Prigge, Claudia Brendler, Falk Liebers, Peter von Löwis, GE,, Andrea Pfeiffer, Falko Papenfuss, Janice Hegewald

**Affiliations:** 1 Division Work and Health, Federal Institute for Occupational Safety and Health (BAuA), Berlin, Germany.; 2 Bosch GmbH, Abstatt/Reutlingen, Germany.

**Keywords:** accelerometry, field study, flexible workplace, hybrid working, physical activity, remote work, sitting, teleworking, working condition

## Abstract

**Objectives:**

Sedentary behavior is associated with diseases (eg, cardiovascular, diabetes type 2). We aimed to describe the sitting and moving behavior of desk-based hybrid employees of a large company in Germany working in either a traditional open plan office (OPO) or an activity-based flex office (AFO) and when working from home. We also aimed to determine if the behaviors differ between both working environments (ie, working from home versus the office) and the office concepts (OPO versus AFO).

**Methods:**

We conducted a cross-sectional study to measure sedentary time, sit-to-stand-transitions (STS), standing, and physical activity (time spent physically active and steps) in different working environments with activPAL3. Time-use data were also examined using compositional data analysis. Mixed model regression was performed to estimate means and 95% confidence intervals (CI). The main models were adjusted for sex, age, profession and measurement phase (July–November 2021).

**Results:**

The sample comprised 102 employees (women: N=27, mean age 38.9 years). On average, OPO employees spent 351 minutes (95% CI 322–380) being sedentary, took 2763 steps (95% CI 2460–3066) and made 16.6 STS (95% CI 13.6–19.6). AFO workers averaged 333 sedentary minutes (95% CI 308–358), 2906 steps (95% CI 2645–3167) and 19.1 STS (95% CI 16.6–21.7). When working from home, workers spent 378 minutes (95% CI 359–396) being sedentary, took 1257 steps (95% CI 1063–1452) and made 20.9 STS (95% CI 19.0–22.8). Working from home was associated with increased sedentary time and fewer steps but more STS.

**Conclusion:**

Sedentary time of desk-based workers seems to be prolonged when working from home. As sedentary behavior increases the risk of disease, there is a need for measures to reduce employees' sedentary time in all working environments.

Sedentary behavior is common among adults. It is defined as sitting, reclining or a lying posture with a low energy expenditure (≤1.5 metabolic equivalent of task) while awake (1). In the European Union, it is estimated that 38.9% of workers spend most of their time at work seated; office workers spend about 70% of their work time and about 66% of their total time awake sedentary (2).

High amounts of sitting contribute to the risk of type 2 diabetes mellitus, cancer, cardiovascular disease, and cardiovascular and all-cause mortality (3–6). Thus, in 2020, the World Health Organization added recommendations on sedentary behavior to their guidelines for physical activity (3). To prevent the negative impact of high amounts of sedentary behavior, time spent sitting should be replaced with physical activity of any intensity or moderate-to-vigorous physical activity should be increased (3, 5, 7). However, meeting the physical activity recommendation alone is not sufficient to counteract the effect of long sitting times (3, 7).

Occupational sedentary behavior is partly determined by environmental factors (eg, office environment) (8–12). One change in the working environment of offices includes the increased use of “activity-based flex office” (AFO) designs (13). AFO contain task-specific areas (eg, for telephone calls, concentrated work, meetings) without a personal desk (8). This may promote healthy behavior, as desk-based employees are expected to move for different tasks (8–12, 14). According to Engelen et al's review (8), the evidence is limited but AFO tend to increase physical activity compared to other office designs. Working from home, which increased during the COVID-19 pandemic will likely continue as an additional working environment (15). This “new normal” also influences the sitting and moving patterns of employees while working and during leisure time. In their systematic review, Wilms et al (16) reported an increase in sedentary behavior when working from home during COVID-19 pandemic restrictions. However, this may not apply to a post-pandemic period, and most studies of the studies in the review used self-reported sitting and physical behavior data (16).

The SITFLEX study was conceptualized to gain information about desk-based workers' sitting and movement in different working environments. It aimed to scientifically evaluate the impact of relocating desk-based workers from a traditional open plan office (OPO) – an open plan office with designated workplace – to an AFO. Due to the start of the COVID-19 pandemic in March 2020, the originally planned interventional study design was changed to a cross-sectional design for better planning certainty. The pandemic also provided the possibility to expand the research questions to include working from home as an additional aspect. Working from home gained importance during the pandemic, and the need for recommendations regarding the design of healthy hybrid work environments remains.

The SITFLEX mixed-method study comprised quantitative (SITFLEX-1) and qualitative (SITFLEX-2) investigations. The latter was performed to identify perceived facilitators and barriers for favorable sitting and moving behavior in the different working environments (17). We aimed to describe the sedentary time in minutes as well as steps and sit-to-stand transitions (STS) of desk-based hybrid OPO or AFO employees in a large company in Germany. We also aimed to assess sitting and moving behaviors when working from home and to determine if these differed when working at the office or between the office concepts (OPO versus AFO).

## Methods

### Study procedure and data collection

**Figure 1:**
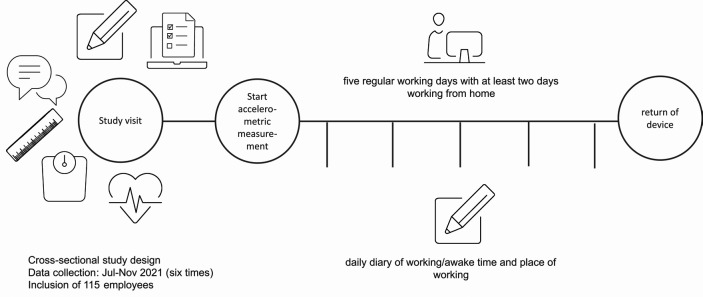
Study procedure.

SITFLEX-1 was a monocentric cross-sectional study including the objective measurement of sedentary behavior and physical activity conducted between July and November 2021 that aimed to gather information about sitting and moving patterns of desk-based workers. It was conducted at one site (with ca. 6500 employees) of an international company in a rural area of Southern Germany. When working on-site, employees worked either in OPO or AFO, but they could also work from home. Most OPO and AFO workstations were equipped with sit-to-stand desks. The AFO had additional areas, such as telephone booths, quiet rooms, spacious open plan rooms, meeting rooms and creativity rooms equipped with flipcharts, large screens and different seating elements. Data collection was performed after the end of lockdown regulations in Germany when working from home was voluntary and the return to on-site working was possible. Participants attended an initial study visit and then their sedentary behavior and physical activity was measured on five consecutive workdays (figure 1).

### Participants

The company was recruited by approaching and gaining the support of managers, staff council members, occupational safety and health services and other representatives. Then, all employees at the location (N=6451) were informed about the study via emails and online information sessions. The on-site medical officer invited employees to participate. Participants received a report of their personal sitting and movement behavior as an incentive. The following inclusion criteria were implemented: desk-based work, age >18 years, ability to stand and walk, planned to work ≥2 days at home and ≥2 days at the company during the measurement phase. Employees were excluded if they were pregnant or had any known skin sensitivities or plaster allergies because the activity monitor was attached to the skin with an adhesive patch.

### Accelerometric measurement and diary

Valid measurements of sitting and moving behavior were obtained using an accelerometer (activPAL3, PAL Technologies, Glasgow, United Kingdom, sampling frequency 20 Hz, software PALconnect) (18). At the study visit, participants were taught how to attach the device to the skin of their right thigh with an adhesive patch and instructed to wear it continuously over five consecutive working days (Monday–Friday). Written instructions based on Edwardson et al (19) were provided to ensure the correct placement of the device. Furthermore, participants documented the times when they were awake and working and where they worked most of the time in a study-specific diary.

### Questionnaires and measurements

Sociodemographic data, smoking status and known high blood pressure were assessed in an interview during the study visit. Blood pressure, height and weight were also measured during the study visit. Afterwards, participants were asked to answer an online questionnaire about their working environments, musculoskeletal disorders and general health. Participation was possible in one of six different measurement phases between July and November 2021.

### Operationalization of the variables

*Outcome variable.* Sedentary time, time spent standing and being physically active, as well as counts of steps and STS during the work time and total time awake were measured objectively with the activPAL3.

Following the definition of Tremblay et al (1), we defined sedentary time as the sum of time spent being sedentary, lying and travelling by car. One STS was defined as the change in activity code from sedentary, lying or travelling by car to standing or stepping.

*Exposure variables.* In an online questionnaire, employees indicated if they worked in an AFO, OPO or “other office concept” when working at the company site (office concept).

In their daily diaries, workers were asked: “Where did you work most of the time today?”. Possible answers were: “at the company”, “mobile work at home”, “other mobile work” (workplace). Days spent with “other mobile work” were excluded from the analysis (N=8).

*Time periods.* Total time awake for every measurement day was identified in the diary by asking participants: “When did you get up?” and “When did you go to bed?”. Data were cross-checked by examining the accelerometric data, and time asleep was not analyzed.

To identify work time, participants were asked to note the start and end of working periods corresponding to the times that would be recorded with a time clock. Lunch breaks were not considered as work time.

*Confounders.* The minimal set of confounders were identified based on directed acyclic graph (20). Participants reported their sex (“male”, “female”, “intersex/transgender”), age in years and their profession [“software engineer”, “hardware engineer”, “admin (controlling, information technology, facility management etc.)”, “employee in sales”, “other: *free text*”]. After examining the free text data for profession, a fifth category for “application engineers” was created. We considered “current profession” in the regression analysis a binary variable differentiating between application engineers and all other professions. This approach was chosen because application engineers have work tasks in laboratories and at desks. Six measurement phases took place between July and November 2021 and were identified by the initial visit date of participants

Operationalization of further variables included in the descriptive analysis is shown in the supplementary material, www.sjweh.fi/article/4228, table S1.

### Data preparation and processing

We combined the diary and the accelerometer data in MatLab Version 14 and identified valid days according to Edwardson et al (19). Further details regarding the processing of the accelerometric data is shown in supplementary figure S1.

As times spent in mutually exclusive behaviors – like sedentary time, standing or physical activity – within a work day are constrained (add up to constant sum, eg, 8-hour workday), they should be analyzed using procedures suitable for constrained data, like compositional data analysis (CODA) (21). CODA uses isometric log-ratio (ILR) transformed data and allows the use of standard statistical methods (21). Therefore, we transformed our data into ILR_1_ and ILR_2_ (21, 22). ILR_1_ corresponds to the ratio of sedentary time (ST) to times spent standing (STAND) and physically active (PA):


ILR1= 23 lnSEDSTAND x PA12 (equation 1)

ILR_2_ correspond to the ratio of time spent standing to time spent physical active:


$${ILR}_{2}=\;\sqrt{\frac{1}{2}}\;ln\frac{STAND}{PA}\;$$ (equation 2)

We calculated ILR_1_ and ILR_2_ for work time and time awake.

### Statistics

The statistical analysis was performed fully syntax based using SPSS statistical software version 29 (IBM Corp, Armonk, NY, USA). Age, sex, and health parameters of the study population were described stratified by office concept, because all study participants also working from home.

We calculated linear mixed models (LMM) estimated with restricted maximum likelihood and random intercepts for study participants. We modelled office concept, workplace and the interaction term (office concept×workplace) as fixed effects and included days of the week as a repeated measurement. Unadjusted mean values and 95% confidence intervals (CI) of sedentary time and time spent standing and being physically active, steps and STS were calculated via post-estimation for work time and total time awake, separately. In the same manner, we estimated work time and total time awake in minutes.

To test for differences between workplace and office concept, we conducted the LMM regression with ILR_1_ and ILR_2_ as dependent variables and the confounders gender, age, application engineer (yes/no) and measurement phase (integers 1–6) as fixed effects. Age and measurement phase were included as mean-centered variables in the regression models.

In the same manner, we estimated the differences in sedentary time, steps and STS between workplaces (working from home – working at the office) and office concepts (OPO-AFO) standardized to an 8-hour work day or a 16-hour day (typical time awake), respectively. For example, to standardize sedentary time during work we divided the sedentary time (minutes) measured during work by the recorded work time (minutes) and multiplied this by the minutes in an 8-hour workday (480 minutes).

## Results

### Study population

A total of 115 employees were recruited and 102 included in the analysis. Reasons for the 13 exclusions were (i) withdrawal from the study (N=1), (ii) failure to complete the online questionnaires (N=3), (iii) not working for ≥2 days from home and at the company (N=3), (iv) and work environment at the company was neither OPO nor AFO (N=6). Number of included days can be found in supplementary figure S2.

Of the participants in the analysis, 26.5% (N=27) were women and 73.5% (N=75) men, and none were intersex/transgender (N=0). They were fulltime employees (median contractual working time: 40 hours per week). The arithmetic mean age was 38.9 years (95% CI 37.0–40.7). The youngest employee included was 20 years and the oldest 60 years. AFO employees were on average younger (37.2 years; 95% CI 34.9–39.5) than OPO employees (41.1 years; 95% CI 38.1–44.2) and were similar with regard to proportion of women and distribution of their current profession (table 1).

**Table 1:** Characteristics of the study population stratified by office concept. [CI=confidence interval]

		Working environments							
		Traditional open plan office			Activity-based flex office			Total ^a^	
		N (%)	Mean (95% CI)		N (%)	Mean (95% CI)		N (%)	Mean (95% CI)
Number of employees per group		43 (42.2)			59 (57.8)			102 (100.0)	
Women		13 (30.2)			14 (23.7)			27 (26.5)	
Mean age (years)			41.1 (38.1–44.2)			37.2 (34.9–39.5)			38.9 (37.0–40.7)
Current profession									
	Software engineer	15 (34.9)			16 (27.1)			31 (30.4)	
	Hardware engineer	<5			<5			7 (6.9)	
	Application engineer	<5			<5			5 (4.9)	
	Administrator	18 (41.9)			28 (47.5)			46 (45.1)	
	Employee in sales	<5			<5			6 (5.9)	
	Others	<5			<5			7 (6.9)	
Self-reported general health (0–10 scale)			7.8 (7.5–8.1)			7.5 (7.1–7.8)			7.6 (7.3–7.8)
Currently not smoking		39 (90.7)				58 (98.3)			97 (95.1)
Normal body mass index (18.5–25 kg/m^2^)		29 (67.4)			31 (52.5)			60 (58.8)	
High blood pressure (anamnestic)		<5			<5			4 (3.9)	
High blood pressure (measured)		<5			Not reported			17 (17.0)	
Limiting disorders in cervical spine		Not reported			<5			8 (7.8)	
Limiting disorders in lumbar spine		6 (14.0)			10 (16.9)			16 (15.7)	

^a^ All workers also worked from home.

### Estimated means during work time

**Figure 2:**
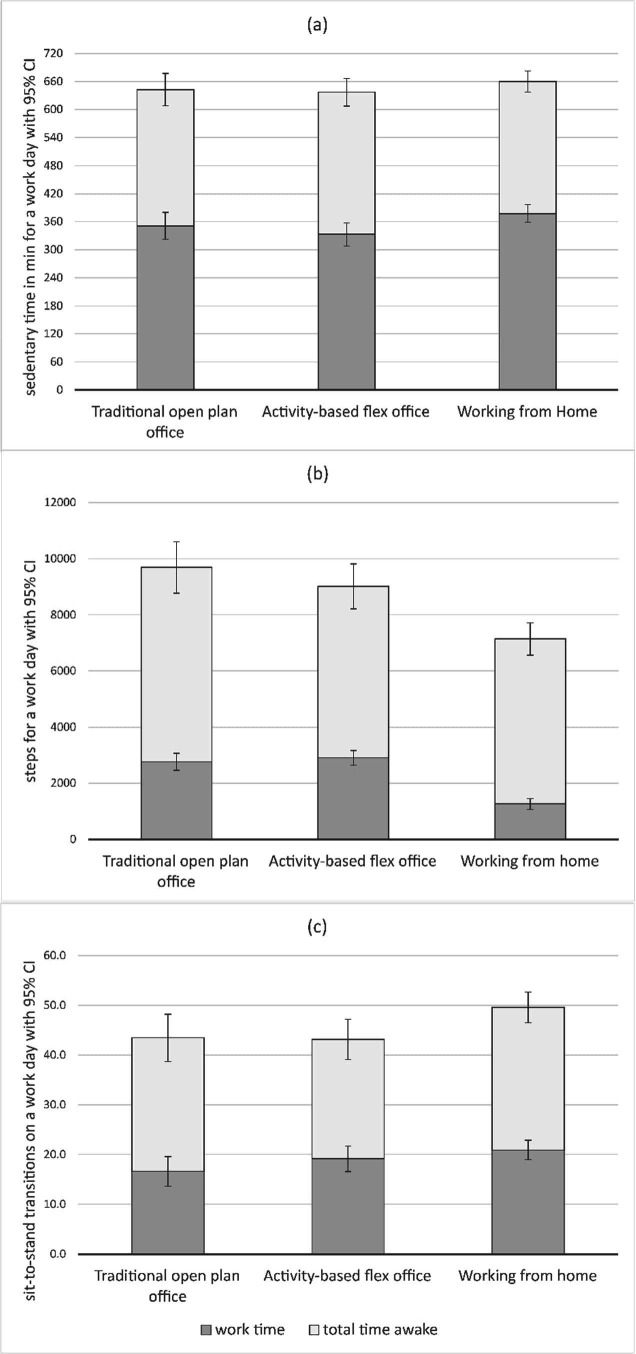
Objectively measured sedentary time (a), steps (b) and sit-to-stand-transitions (c) stratified by working environments during working and total time awake with 95% confidence interval (CI).

During work time (not standardized to 8 hours), OPO and AFO employees were sedentary an average of 351 minutes (95% CI 322–380) and 333 minutes (95% CI 308–358), respectively, compared to 378 minutes (95% CI 359–396) when working from home (figure 2a). Means and 95% CI of time spent standing and physically active are shown in table 2.

**Table 2:** Time in minutes spent sedentary, standing and physically active in different environments. [CI=confidence interval]

		Traditional open plan office		Activity-based flex office		Working from home
		Mean (95% CI)		Mean (95% CI)		Mean (95% CI)
Work time		493 (474–513)		495 (478–512)		480 (467–493)
	Sedentary	351 (322–380)		333 (308–358)		378 (359–396)
	Standing	110 (87–132)		123 (104–142)		78 (64–93)
	Physical activity	30 (27–34)		32 (29–35)		18 (16–21)
Total time awake		987 (973–1001)		992 (980–1004)		972 (963–981)
	Sedentary	643 (608–677)		637(608–667)		660 (637–682)
	Standing	234 (205–264)		248 (223–274)		217 (198–236)
	Physical activity	109 (100–119)		103 (94–111)		90 (84–97)

OPO employees averaged 2763 steps (95% CI 2460–3066), and AFO employees 2906 steps (95% CI 2645–3167), and 1257 steps (95% CI 1063–1452) while working from home (figure 2b).

Considering the number of STS during work time, we found that OPO employees interrupted their sitting on average 16.6 times (95% CI 13.6–19.6, ca. 2.1 times/hour) and AFO employees averaged 19.1 STS (95% CI 16.6–21.7, ca. 2.4 times/hours). When working from home, participants averaged 20.9 STS (95% CI 19.0–22.8, ca. 2.6 times/hour) during work time (figure 2c).

### Estimated means during total time awake

Considering total time awake, OPO and AFO employees, spent 643 (95% CI 608–667) and 637 (95% CI 608–677) minutes sedentary, respectively compared to 660 (95% CI 637–682) minutes when working from home (figure 2a). Means and 95% CI of time spent standing and physically active in minutes for total time awake are shown in table 2.

Considering steps during total time awake, OPO employees averaged 9694 (95% CI 8779–10 609) and AFO employees averaged 9015 (95% CI 8215–9814) steps versus 7140 (95% CI 6563–7717) steps when working from home (figure 2b).

During total time awake, OPO and AFO employees, interrupted their sitting with an average of 43.4 (95% CI 38.7–48.2) and 43.1 (95% CI 39.1–47.2) times, respectively versus 49.6 (95% CI 46.5–52.7) times when working from home (figure 2c).

### Estimated differences between working from the office versus home and office concepts during work time

**Figure 3:**
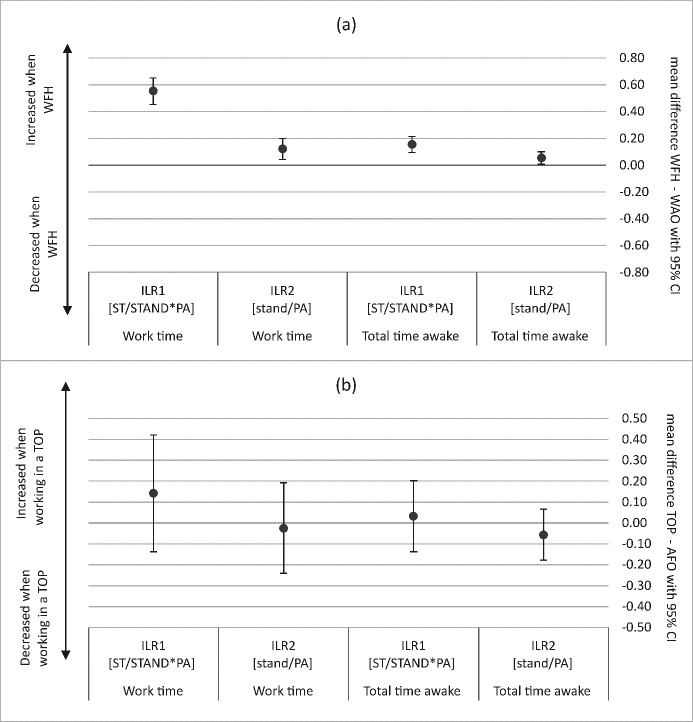
Mean difference with 95% confidence interval (CI) in isometric log-ratio (ILR_1_ and ILR_2_) (a) between working from home (WFH) and working at the office (WAO) and (b) between traditional open plan office (OPO) and activity-based flex office (AFO). [ST=sedentary time; STAND=time spent standing; PA=time spent physically active.]

After adjustment for sex, age, work as an application engineer and measurement phase, analysis of the compositional data showed that sedentary time increased relative to time spent standing and being physically active (ILR_1_), and standing time increased relative to time spent physically active (ILR_2_) when working from home (figure 3). Post-estimation of the LMM with sedentary time standardized to an 8-hour workday estimated an increase in sedentary time of 46 minutes (95% CI 36–56) (figure 3a). Over an 8-hour workday, the number of steps decreased by 1487 (95% CI -1658– -1317) (figure 3b) and the number of STS increased by 3.5 (95% CI 2.1–5.0) when working from home compared to the office (figure 3c). No difference between the office concepts could be found as shown in figures 3 and 4.

### Estimated differences between working from the office versus home and office concepts during total time awake

When working from home, sedentary time increased relative to time spent standing and being physically active (ILR_1_), and time spend standing relative to time spent with physical activity increased (ILR_2_) compared to office-based work (figure 3). LMM post-estimation of the sedentary time (standardized to 16 hours) estimated the increase when working from home to be 31 (95% CI 17–46) minutes during total time awake (figure 4a).

Using the LMM post-estimation, we found that steps were reduced by 2020 (95% CI -2608– -1432) (figure 4b), and workers interrupted their sitting more while awake (16 hours) (7.0 STS, 95% CI 4.8–9.2) (figure 4c) on days working from home versus the office.

No difference between the office concepts could be found during total time awake as shown in figures 3 and 4.

**Figure:**
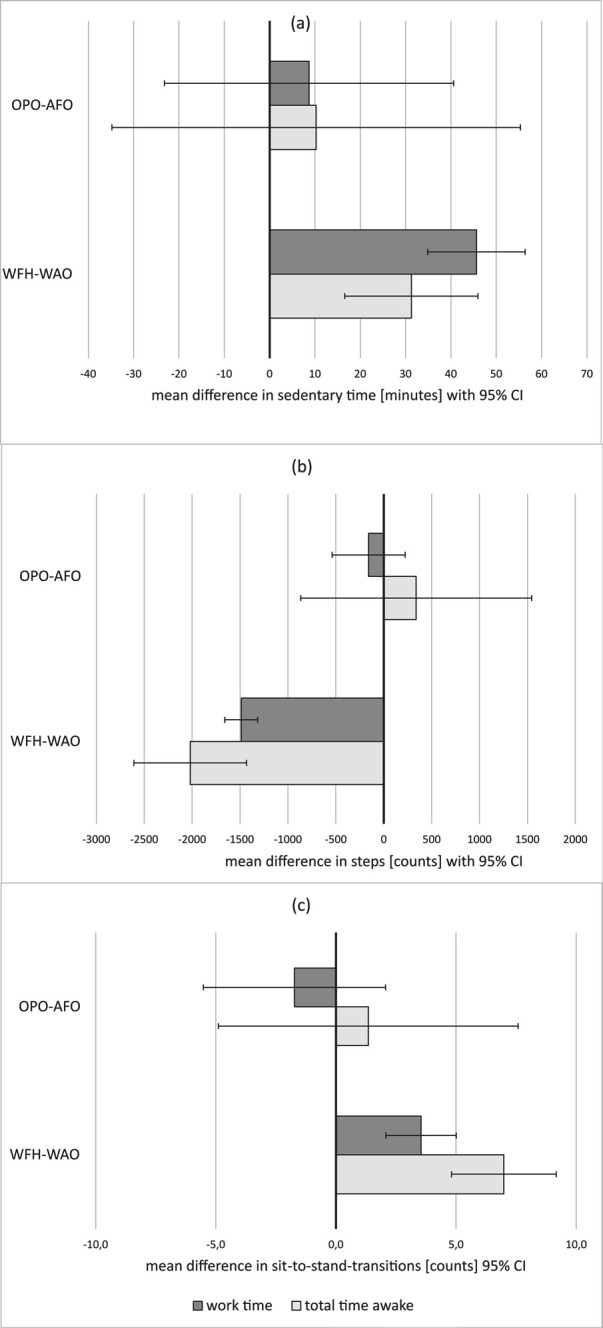
**Figure 4.** Differences in sedentary time (in minutes) (a), count of steps (b) and sit-to-stand-transitions (c) during work time and total time awake with 95% confidence interval (CI). [WFH=working from home; AFO= activity-based flex office; OPO=traditional open plan office].

## Discussion

We found differences in sitting and moving behaviors of desk-based workers related to workplace. Longer sedentary time, fewer steps, and more STS were observed during work time when employees worked at home compared to at the office. Considering total time awake, desk-based workers also sat longer and made fewer steps but performed more STS on days working from home compared to the office.

High overall sitting times were prevalent among hybrid office workers independent of the workplace or office concept (>5.5 hours during work time and >10.5 hours during total time awake). When working from home, the desk-based workers sat 46 minutes longer while working and about half an hour longer over the whole day.

These results are in accordance with other studies among office workers for the entire day (9.5–10.5 hours per day) (23, 24) and for work time (over 6 hours) (25, 26). Furthermore, Thralls Butte et al (23) and Holmes et al (25), using objectively measured data of office workers, also reported increases in sedentary behavior when working from home compared to at the office (23, 25). On the other hand, Hallman et al (26) found no relevant difference in the distribution of objectively measured sedentary and active behavior of office workers over a day between working from home and at the office, but more sleep on days working from home. Also, Kim et al (27), Sers et al (28) and Wahlström et al (24) found no significant differences in objectively measured sedentary behavior among office workers when comparing working from home to working at the office.

On average, employees in this study took about 7200 and >9000 steps per day working from home and the office, respectively, during total time awake. These values are slightly higher than the values of office workers observed by Sers et al (28), who reported 6299 steps (SD 3779) and 7548 steps (SD 3944) when working from home and at the office, respectively. During work time, employees in this study walked about 2800 to 2900 steps at the office and about 1300 steps when working from home. The high number of steps walked at the office could be due the extensive grounds of the worksite.

After adjustment for confounders, a reduction of steps was found when working from home during work time (about 1500 fewer steps) and during total time awake (about 2000 fewer steps). This result is in accordance with the results of Holmes et al (25), Kim et al (27) and Sers et al (28), all of whose studies included only offices workers. Holmes et al (25) reported fewer steps during work when working from home compared to at the office. Also, Kim et al (27) and Sers et al (28) found fewer steps for the entire day when working from home. On the other hand, Thralls Butte et al (23) reported more steps of desk-based workers working from home exclusively compared to on-site office workers.

One explanation for longer sitting times while working from home was found in the qualitative interview study (SITFLEX-2) (17). Employees reported using the home environment for concentrated work, which is often performed in a sitting position. The reduction of steps during work time when working from home could also be explained by shorter distances to the bathroom or kitchen. Another explanation for this difference may be a divergence in the documentation of work time between workplaces. It may be harder to differentiate between work and leisure time when working from home. For example, the start of work at home was documented when sitting at a desk, while the on-site workday began by checking in at the time clock. Considering the difference of steps during total time awake, commuting – one potential contributor to physical behavior (29) – probably did not explain the difference as employees usually drive a car to the rural office location, and the difference remained for steps made during work time.

On average, employees interrupted their sitting >2 times per hour while working (OPO, AFO and working from home: 2.1, 2.4 and 2.6 times per hour, respectively), which lies formally in the recommended range of the European Agency of Safety and Health at Work for the frequency of micro-breaks (20–30-second breaks, including stretching) (30). However, our results are the average numbers of breaks over work time. This means these may not be evenly distributed, and there might be peak times for interrupting sitting with breaks (eg, at the beginning or shortly before the end of work). Furthermore, we defined sitting interruptions as changing from a sitting to standing position or walking without any stipulation for the minimum required time in the non-sitting position. This means that the number of sitting breaks, which can have an impact on the metabolism (2–5 minutes) (31, 32), may be lower (33).

Considering the number of STS over the whole day, employees tended to interrupt their sitting more often when working from home. Widar et al (34) reported more STS during work time at home compared to a conventional workplace (34). Results of the interview study (SITFLEX-2) indicated that this could be explained by the need for short sitting interruptions at home due to private issues (eg, opening the door for the postman) or increased freedom to stretch or move than at the office (17). In contrast, Holmes et al (25) found more STS of workers during the work time when working at the office (25).

No differences could be found between the two on-site office concepts during work time. This could be due to pandemic-related utilization levels of the offices and because some desks were designated unavailable to prevent the spread of infection. Conditions were still exceptional during our study and not the typical conditions for which the AFO-concept was devised. Therefore, the impact of the design differences between the office concepts may have been lessened during our study. Furthermore, the statistical power is limited due to relatively small groups for the office concepts. Thus, these results should be regarded with caution. For example, Wahlström et al (14) observed increase physical movement in AFO compared to cell offices, but no change of sedentary time (14). Hallman et al (10) found heterogeneous results across different office sites with a decrease in sitting time ranging from +1.4– -18.3%, thus no overall effect was found (10). According to Hallman et al (10), this might be due to site-specific determinants influencing the improvement of sedentary behavior and physical activity when AFO are implemented.

The sedentary time and step count differences between working from home versus at the office may impact health. A risk reduction for all-cause mortality can be achieved by prolonging moderate-to-vigorous physical activity (35), but physically active time decreases when sedentary time increases. Furthermore, the step reductions we observed are health-relevant. Del Pozo Cruz et al (36) find a negative association between an increase of about 2000 steps and all-cause mortality, cardiovascular incidence/mortality and cancer incidence/mortality (36).

Among the strengths of this study was the study population. We measured the sedentary and physical activity behavior of >100 employees. Furthermore, we obtained data measured objectively via an accelerometric sensor. We can differentiate between work and leisure time, which provides a holistic impression of the sedentary and physical activity behavior of desk-based workers.

Our study has some limitations. The study only included employees from one company site. Measurement phases started shortly after the end of lock-down regulations due to COVID-19 pandemic in July 2021. Employees of the company had not yet fully returned to on-site work in a regular manner, and some preferred working from home after the pandemic restrictions were lifted. This could be a reason for the low participation rate, as the study participation required presence at the office ≥2 days of the week. Also, the on-site work requirement might have increased the self-selection of health-conscious participants interested in the study topic. While the low participation and self-selection may have caused selection bias, the characteristics of participants reflected that of the company site with regard to age and sex. Also, sedentary and physical activity behavior may be influenced by the fact that the mandate to work from home had just ended and some behaviors may still have been influenced by measures to prevent infections.

### Concluding remarks

In this study, we found working from home increased sedentary behavior relative to time spent standing and being physically active among hybrid desk-based employees of a large German company. We could not detect differences between AFO and OPO employees’ sitting and moving behaviors, but this could be due to the exceptional post-pandemic situation. There is a need for further research into the new and persisting working conditions created by the pandemic, such as the health implications of contemporary office concepts and working from home. Companies need guidelines to help their employees reduce their overall sitting time whether working at the office or at home as sedentary behavior increases the risk of disease.

## Supplementary material

Supplementary material

## Protection of research participants

The Ethics Committee of the medical association of Berlin (Eth-24/21, April 19, 2021) approved the study. All participants gave their written informed consent and were informed about the study previously. Concept of the data protection was approved by the official data protection officer of the Federal Institute for Occupational Safety and Health (BAuA) (confirmation received 24.02.2021). Data collection was pseudonymized, which means that identification was only possible with a code list. This list was destroyed after sending personal feedback to all participants of a measurement phase.
