# Supplementary material for: Comparison of sedentary time, number of steps and sit-to-stand-transitions of desk-based workers in different office environments including working from home: analysis of quantitative accelerometer data from the cross-sectional part of the SITFLEX Study
Source: Scand J Work Environ Health. 2025 Jun 26;51(4):333–43. doi: 10.5271/sjweh.4228 (PMC12282600; doi:10.5271/sjweh.4228)
Supplement: Supplementary material [file SJWEH-51-333-S001.pdf]

Comparison of sedentary time, number of steps and sit-to-stand-transitions of desk-based workers in different office environments including working from home: analysis of quantitative accelerometer data from the cross-sectional part of the SITFLEX Study[1]

*by Martha Sauter, MPH, Eva Backé, PhD, Carina Pfab, MScPH, Michaela Prigge, MScPH, Claudia Brendler, PhD, Falk Liebers, PhD, Peter von Löwis, GE, Andrea Pfeiffer, PhD, Falko Papenfuss, PhD, Janice Hegewald, PhD* [2]

1. Supplementary material
2. Correspondence to: Janice Hegewald, Division Work and Health, Federal Institution for Occupational Safety and Health (BAuA), Nöldnerstr. 40-42, 10317 Berlin, Germany [E-Mail: Hegewald.Janice@baua.bund.de]

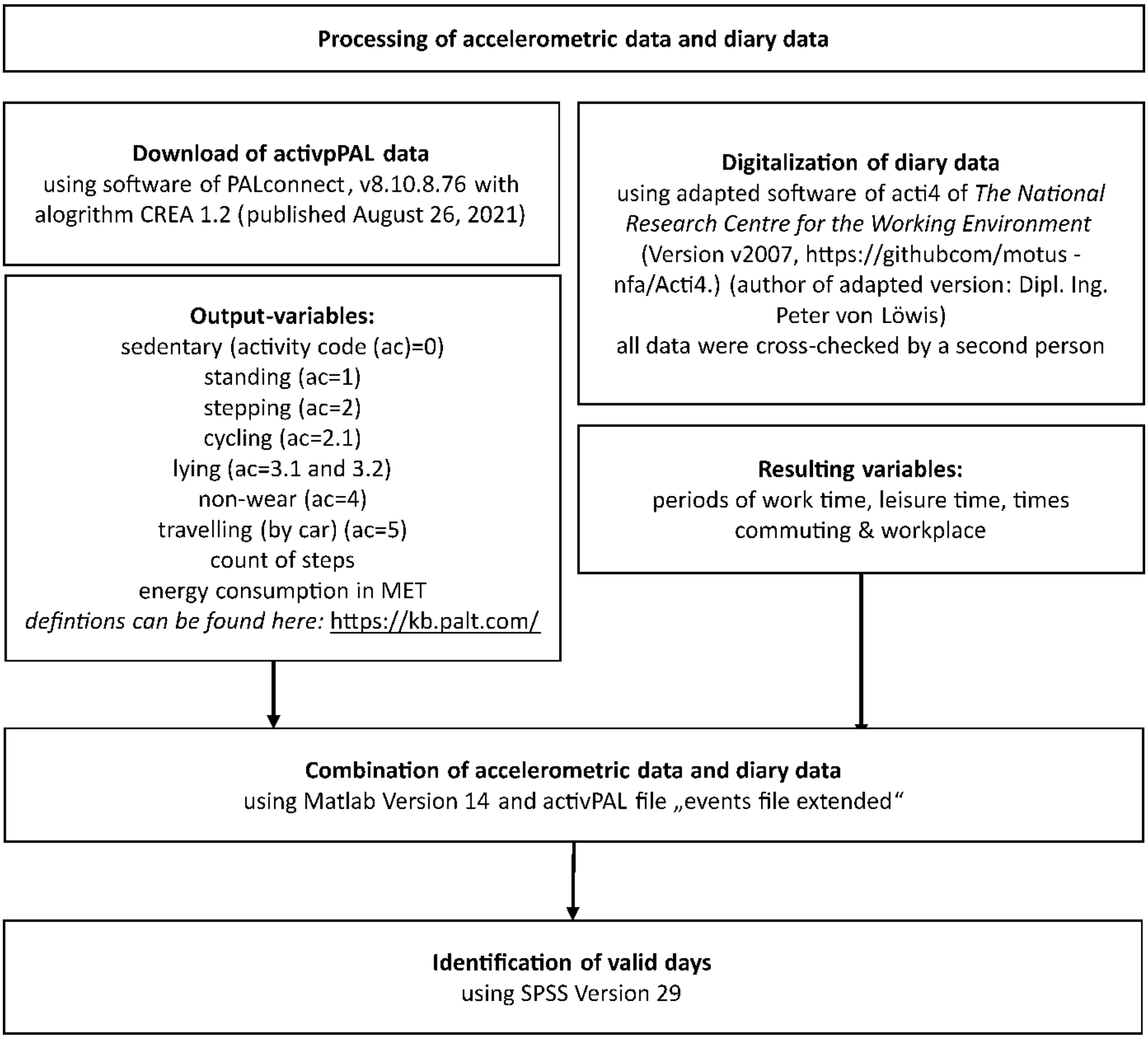


Supplementary figure S1. Processing of accelerometric data and diary data

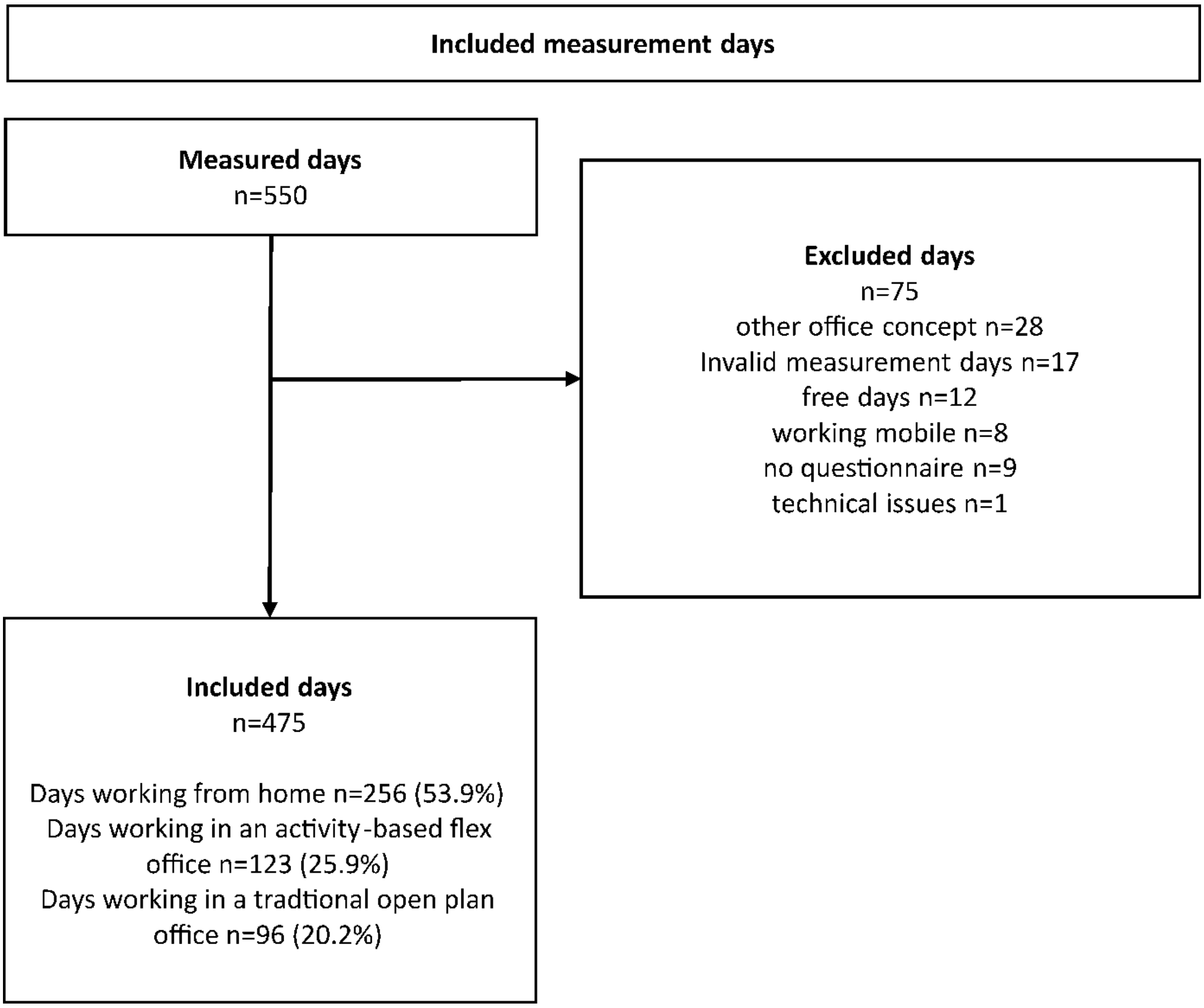


Supplementary figure S2. Development of included measurement days

Supplementary table S1. Further information about operationalization of variables

| Variables | Used Questionnaire/Measurement method | Definitions/Question and answers | Proceeding |
|---|---|---|---|
| **Sedentary time *(Outcome variable)*** | activPAL3 *(accelerometric measurement)* | Sedentary (ac=0) + lying (ac: 3.1 and 3.1, during total time awake) + traveling (by car) (ac=5) | - |
| **Sit-to-stand-transition *(Outcome variable)*** | activPAL3 *(accelerometric measurement)* | the change in activity code from sedentary (ac=1), lying (ac=3.1 and 3.2, during total time awake) or travelling (by car) (ac=5) to standing (ac=1) or stepping (ac=2). | - |
| **General health** | Copenhagen Psychosocial Questionnaire (COPSOQ) | Participants were asked to rate their general health with a number between 0 and 10 | - |

| | | | |
|---|---|---|---|
| ***(Descriptive variable)*** | | (10 relates to a very good health status). | |
| **Smoking status** ***(Descriptive variable)*** | According to Robert Koch Institute | "Do you smoke?" Possible answers (single choice):"yes, every day", "yes, sometimes", "no, I stopped" or "no, I never smoked". | The number and percentage of non-smokers were identified by adding up the categories "no, I stopped" and "no, I never smoked" |
| **Known blood pressure** ***(Descriptive variable)*** | According to Robert Koch Institute | "Have you ever been diagnosed with high blood pressure by a doctor?", "Did you have high blood pressure in the last 12 months?", "Are you currently taking antihypertensive medication?" | - |
| **Limiting disorders of the cervical and lumbar spine** ***(Descriptive variable)*** | German Version of the Nordic Musculoskeletal Questionnaire | "Did you have pain in lumbar/cervical spine the last four weeks?" Possible answers: "yes" or "no" If "yes", the following was asked: "Did this pain limit you in the last four weeks during your work or in your leisure time?" Possible answers: "yes" or "no" | Information of the two questions were combined, resulting in a variable that indicated if an employee had limiting pain in their [lumbar/cervical] spine in the last four weeks ("yes" or "no"). Employees who had no pain or no limiting pain were categorized into "no". |
| **Measured blood pressure** ***(Descriptive variable)*** | In seated position three times during the study visit after at least 10 minutes of rest and at least 2 minutes time between the measurements with the device X7 Smart (Omron) | - | Mean values of systolic and diastolic blood pressures was calculated |
| **Height** | Measured without shoes with two decimals in meter with and Stadiometer (Seca 213) | - | - |
| **Weight** | Measured without shoes and in light clothing to one decimal place in kilograms with an electronic scale (Soehnle) | - | - |
| **Body Mass Index** ***(Descriptive variable)*** | Calculated with measured height and weight according to World Health Organization (WHO) | - | - |

Supplementary table S2. Estimated means of sedentary behavior and steps – data corresponding to figure 2

| | OPO | AFO | WFH |
|---|---|---|---|
| | Mean (95% CI) | Mean (95% CI) | Mean (95% CI) |
| **Work time** | | | |
| **sedentary time in min** | 351 (322–380) | 333 (308–358) | 378 (359–396) |
| **steps n** | 2763 (2460–3066) | 2906 (2645–3167) | 1257 (1063–1452) |
| **STS n** | 16.6 (13.6–19.6) | 19.1 (16.6–21.7) | 20.9 (19.0–22.8) |
| **Total time awake** | | | |
| **sedentary time in min** | 643 (608–677) | 637 (608–667) | 660 (637–682) |
| **steps n** | 9694 (8779–10 609) | 9015 (8215–9814) | 7140 (6563–7717) |
| **STS n** | 43.4 (38.7–48.2) | 43.1 (39.1–47.2) | 49.6 (46.5–52.7) |

**Results are adjusted for gender, age, current profession and measurement phase and standardized of outcome variables to an eight-hour working day and a 16-hour day, respectively**.

**Abbreviations: AFO – activity-based flex office; CI – confidence interval; min – minutes, n – number; STS – sit-to-stand-transitions; OPO – traditional open plan office; WFH – working from home**

Supplementary table S3. Mean difference between working from home and working at the office with 95% confidence interval for ILR1 und ILR2 and for OPO and AFO – data corresponding to figure 3.

| | $ILR_1$ | $ILR_2$ |
|---|---|---|
| | Mean difference (95% CI) | Mean difference (95% CI) |
| **Work time** | | |
| **WFH-WAO** | 0.55 (0.45–0.65) | 0.12 (0.04–0.20) |
| **OPO-AFO** | 0.14 (-0.14–0.42) | -0.02 (-0.24–0.19) |
| **Total time awake** | | |
| **WFH-WAO** | 0.15 (0.09–0.21) | 0.05 (0.01–0.10) |
| **OPO-AFO** | 0.03 (-0.14–0.20) | -0.06 (-0.18–0.07) |

**Abbreviations: AFO – activity-based flex office; CI – confidence interval; $ILR_1$ – Isometric log-ratio 1 (SB/Stand and PA); $ILR_2$ – Isometric log-ratio 2 (STAND/PA); OPO – traditional open plan office; WAO – working at the office; WFH – working from home**

Supplementary table S5. Estimated difference in workplace and office concepts – data corresponding to figure 4

| | OPO vs. AFO (ref.) | WFH vs. WAO (ref.) |
|---|---|---|
| | Mean (95% CI) | Mean (95% CI) |
| **Estimated difference during work time** | | |
| **Δ sedentary time in min** | 9 (-23–41) | + 46 (35–56) |
| **Δ steps n** | -159(-540–223) | -1487 (-1658– -1317) |
| **Δ STS n** | -1.7 (-5.5–2.1) | 3.5 (2.1–5.0) |
| **Estimated difference during total time awake** | | |
| | Mean (95% CI) | Mean (95% CI) |
| **Δ sedentary time in min** | +10 (-35–55) | +31 (17–46) |
| **Δ steps n** | 337 (-867–1542) | -2020 (-2608– -1432) |
| **Δ STS n** | 1.3 (-4.9–7.6) | 7.0 (4.8–9.2) |

**Results are adjusted for gender, age, current profession and measurement phase and standardized of outcome variables to an eight-hour working day and a 16-hour day, respectively.**

**Abbreviations: AFO – activity-based flex office; CI – confidence interval; min – minutes; n – number; OPO – traditional open plan office, WAO – working at the office WFH – working from home; STS – sit-to-stand-transitions; Δ – delta**

Supplementary table S6. Unadjusted estimated difference between working environments

| | OPO vs. AFO (ref.) | WFH vs. WAO (ref.) |
|---|---|---|
| | Mean (95% CI) | Mean (95% CI) |
| **Estimated difference during work time** | | |
| **Δ sedentary time in min** | 18 (-20–56) | 36 (21–50) |
| **Δ steps n** | -143 (-543–257) | -1577 (-1752– -1402) |
| **Δ STS n** | -2.5 (-6.4–1.4) | 3.0 (1.5–4.5) |
| **Estimated difference during total time awake** | | |
| | Mean (95% CI) | Mean (95% CI) |
| **Δ sedentary time in min** | 6 (-40–51) | 20 (4–36) |
| **Δ steps n** | 679 (-536–1894) | -2214 (-2817– -1611) |

| | | | |
|---|---|---|---|
| | **Δ STS n** | 0.3 (-6.0–6.5) | 6.3 (4.0–8.6) |

**abbreviations: AFO – activity-based flex office; CI – confidence interval; min – minutes; n – number; OPO – traditional open plan office; WAO – working at the office; WFH – working from home; STS – sit-to-stand-transitions; Δ – delta**
